# The Cæsarian Operation Considered in a Theological Point of View

**Published:** 1847-01

**Authors:** 


*The Cæsarian Operation considered in a Theological point of view.*—Some months since a woman died in Brittany, being about six months advanced in pregnancy. The parish priest sent for a medical man to remove the child by the Cæsarian section, in order that it might be baptised, if still alive. Upon his refusing to do so, the priest sent for a *farrier* who removed a dead fœtus by the Cæsarian section. These facts being severely commented upon in some of the newspapers, the minister published a reply in justification of his proceedings, stating that they were imperative upon him, in proof of which he quoted Bouvier's *Sacred Embryology*, which is a text-book of the church. This expressly orders the section to be made as speedily as possible, under all circumstances as regards the age, time, and mode of death, &c., and in respect to the person who is to be called upon, it says:—"No endeavor must be neglected to procure the services of a professional man. If this is impossible, a midwife, some other woman, a married man, or in case of urgency any one at hand must be resorted to, but *never a priest*, unless there is absolutely no other person who can be procured." When the medical man refused, the priest read these and other passages of M. Bouvier's work to the farrier, showing him the absolute necessity of proceeding with the operation.

A medical man, before resorting to the operation, has to satisfy himself—1, That the *woman is really dead;* 2, that the *child is alive;* and 3, that it is *viable.* This restricts obviously the operation within very narrow limits. The Church regards not the *viability* of the child, but the chance of its being alive, and therefore fitted for baptism. But the chances of error and danger that this rule gives rise to are obvious. Is the practitioner to submit to the dictates of the priest, and open the woman's body the instant she has yielded her last breath. Many there are who would hesitate to do so, and if the professional man delays in order to be certain that his patient is really dead, does it seem reasonable to allow persons utterly destitute of physiological knowledge to take upon themselves not only to judge of the propriety of the operation, but even its execution? This has been so strongly felt by some of the clergy themselves, even, that various contrivances have been thought of to dispense with the necessity of the operation, and yet confer the rite of baptism upon the infant. Thus, *vagino-uterine injections* have been advised, and quite recently, these have given rise to a learned discussion in the Academy of Medicine of Belgium. The validity of such a procedure has been admitted by high authorities of the church, so that, even according to its own showing, the nessity for the operation may be brought within very narrow limits. When, however, it is determined to practice the operation, this should be solely confided to professional men; for an apparent state of death during gestation, is of too frequent occurrence, and of too difficult detection, to allow of the interference of unskilful persons. When medical men are regularly consulted in these cases, they should bear in mind the duties and obligations of their profession, and lend their assistance, when the rights of humanity, of which they are the natural guardians, do not oppose, to the accomplishment of the duties of religion, in the same manner as they are accustomed to do with regard to those of justice.—*Gaz. Medicale.* —*Med. Chir. Rev.*

